# Light-Addressable Potentiometric Sensor as a Sensing Element in Plug-Based Microfluidic Devices

**DOI:** 10.3390/mi7070111

**Published:** 2016-07-01

**Authors:** Ko-Ichiro Miyamoto, Takuya Sato, Minami Abe, Torsten Wagner, Michael J. Schöning, Tatsuo Yoshinobu

**Affiliations:** 1 Department of Electronic Engineering, Tohoku University, 6-6-05 Aza-Aoba, Aramaki, Aoba-ku, Sendai 980-8579, Japan; t.sato.tohoku@gmail.com (T.S.); minami.abe.1190@gmail.com (M.A.); nov@ecei.tohoku.ac.jp (T.Y.); 2 Institute of Nano- and Biotechnologies, Aachen University of Applied Sciences, Heinrich-Mußmann-Str. 1, Jülich 52428, Germany; torsten.wagner@fh-aachen.de (T.W.); schoening@fh-aachen.de (M.J.S.); 3 Peter-Grünberg Institute (PGI-8), Research Centre Jülich, Jülich 52425, Germany; 4 Department of Biomedical Engineering, Tohoku University, 6-6-05 Aza-Aoba, Aramaki, Aoba-ku, Sendai 980-8579, Japan

**Keywords:** chemical sensor, plug-based microfluidic device, light-addressable potentiometric sensor

## Abstract

A plug-based microfluidic system based on the principle of the light-addressable potentiometric sensor (LAPS) is proposed. The LAPS is a semiconductor-based chemical sensor, which has a free addressability of the measurement point on the sensing surface. By combining a microfluidic device and LAPS, ion sensing can be performed anywhere inside the microfluidic channel. In this study, the sample solution to be measured was introduced into the channel in a form of a plug with a volume in the range of microliters. Taking advantage of the light-addressability, the position of the plug could be monitored and pneumatically controlled. With the developed system, the pH value of a plug with a volume down to 400 nL could be measured. As an example of plug-based operation, two plugs were merged in the channel, and the pH change was detected by differential measurement.

## 1. Introduction

Microfluidic devices are advantageous in various aspects for biological or clinical assays where it is necessary to handle small-volume samples. Common processes such as filtering, mixing, separating, heating, cooling, and sensing of the final products after a series of steps are realized by microfluidic systems constructed on a small substrate known as Micro-TAS or Lab-on-a-chip. They are expected to reduce the cost by minimizing the amount of required samples and reagents.

In pursuit of the volume advantage of microfluidic devices, it is reasonable to divide the sample solution into small volumes and manipulate them in the form of plugs or droplets [1,2,3,4]. Consumption of reagents can be saved by reducing the volume, while a plug or a droplet can act as an independent reaction chamber by itself. To realize such a system, a sensing element is required that can probe a plug or a droplet inside the channel.

In this study, we propose a plug-based microfluidic device based on a light-addressable potentiometric sensor (LAPS [5]), which is a chemical sensor based on the field effect in a semiconductor. In our previous papers, we applied a LAPS for measurement of continuously flowing samples in a microfluidic device [6,7], which featured (1) a complete flat sensor surface due to a simple structure consisting of silicon, insulator and electrolyte; (2) a flexible definition of measurement areas by illumination; and (3) the label-free detection and visualization of chemical species. It was also possible to visualize the spatial distribution of ions inside the flow [8]. In the case of continuously flowing samples, however, the consumption of the solution was still large, which spoiled the main advantage of microfluidic devices. To solve this problem, a plug-based version of a microfluidic device combined with LAPS is developed, which can (1) control the position of a plug in the channel; (2) mix two plugs for reaction; and (3) detect the change by differential measurement.

## 2. Experiments

**Figure 1:**
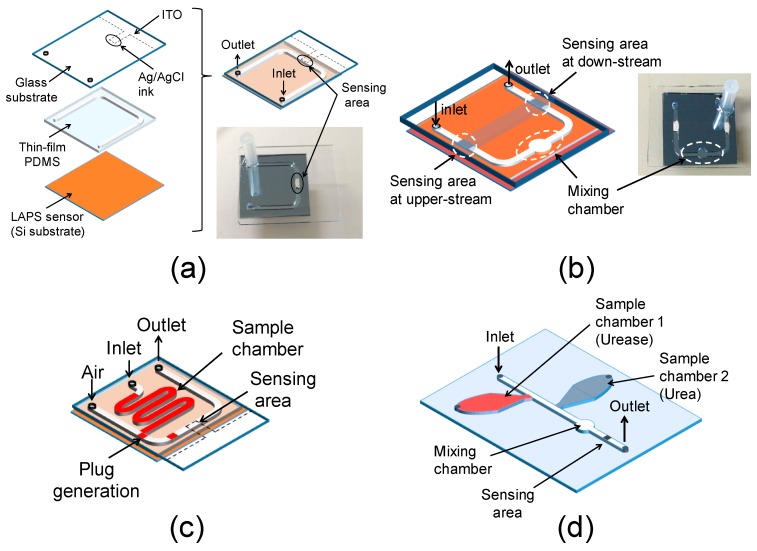
(**a**) Test structure of the microfluidic device combined with LAPS; (**b**) Channel design with a chamber for merging and differential measurement; (**c**) Channel design to generate plugs on chip; (**d**) Test structure with two sample chambers, one merging chamber, and one sensing area.

A microfluidic channel on the sensing surface of the LAPS is depicted in Figure 1a. It consists of a LAPS sensor plate, polydimethylsiloxane (PDMS) film and a glass cover with an Ag/AgCl electrode.

LAPS: The sensor substrate was n-type silicon with a size of 18 mm × 18 mm and a thickness of 200 μm, insulated with a thermal oxide layer and a silicon nitride layer deposited by LP-CVD [6,7,8].

PDMS film: A 500-μm-thick PDMS film to define the channel pattern was prepared by casting the PDMS (Silpot 184, Dow Corning Toray Co., Ltd., Tokyo, Japan) in a polished aluminum mold, which was pressed and cured by heating. The width and the height of the U-shaped channel defined by the PDMS film were 1 mm and 500 μm, respectively.

Glass cover and assembly: To allow optical observation of a plug in the channel, a glass cover was used as the ceiling of the channel, on which an Ag/AgCl electrode was prepared by the following process. Firstly, the electrode pattern was defined by a masking tape on an indium tin oxide (ITO)-coated glass plate, and unnecessary ITO was removed by etching with HCl after application of the mixture of zinc powder and glycerin (3 g/mL). Then, the patterned PDMS thin film was bonded onto the glass cover and Ag/AgCl ink for the reference electrode (No. 011464, ALS Co., Ltd., Tokyo, Japan) was painted to cover the ITO pattern inside the channel. The width, the length and the thickness of the Ag/AgCl pattern were 1 mm, 2 mm, and about 70 μm, respectively. Finally, the other side of the PDMS film was bonded onto the sensor surface after opening the inlet and outlet by an ultrasonic drill. Prior to each bonding process, the surfaces were treated by O_2_ plasma under a pressure of 0.4 mbar with a power of 10 W for 1 min using a plasma cleaner (Zepto, Diener electronic GmbH, Ebhausen, Germany). The bonding was finalized by heating at 95 °C for 10 min. If an optical observation of the plug is not necessary, the whole area of the ceiling may be covered with Ag/AgCl so that the measurement can be done at any point inside the channel. In this case, the measured points are defined by light beams that induce a photocurrent dependent on the local value of pH.

Figure 1b shows another channel pattern with a wider portion proposed by Itoh et al. [9], in which two successive plugs can be merged. In this structure, the first plug remains inside until the second plug arrives, which is merged with the first plug and leaves the chamber together. Two measurement areas are defined by Ag/AgCl electrodes in the upstream and the downstream of the chamber, which allow differential measurement of the pH change. In this study, this configuration was applied to a measurement of enzymatic reactions, where the two plugs contained an enzyme and its substrate, respectively.

Figure 1c shows a channel pattern with a sample chamber. The chamber acts as a dispenser, which divides the sample solution into a series of plugs. The sample solution in the sample chamber was pushed into the main channel by air from a syringe pump, and another continuous air flow in the main channel repeatedly split the injected sample every time the main channel was occluded. Figure 1d shows an integrated structure of two sample chambers, one merging chamber and one measurement point in the downstream, which was examined in this study.

**Figure 2:**
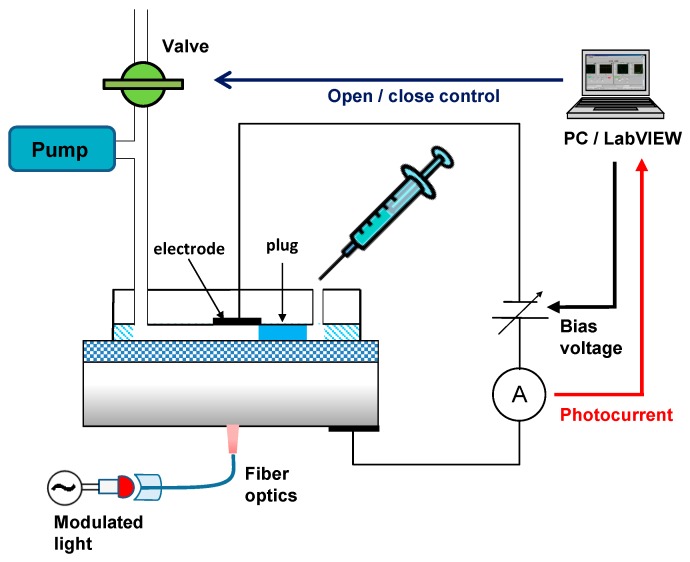
Schematic view of the measurement system.

Measurement setup: The measurement setup used in this study is depicted in Figure 2. A droplet of sample solution is supplied at the inlet using a micro-syringe (SGE analytical science, Victoria, Australia) and sucked into the channel as a plug by a peristaltic pump (AC-2120, ATTO Corp., Tokyo, Japan) when the vent valve (PM-0815W, Takasago Electric Inc., Nagoya, Japan) in the downstream is closed. The position of the plug is controlled by opening/closing the vent valve. The line velocity of the plug was 0.78 mm/s by aspiration of the pump. The sensing area of the LAPS is addressed by illumination of a modulated light from a red-colored LED guided by an optical fiber (ϕ = 1 mm). The photocurrent signal is amplified by a transimpedance amplifier (10^6^ V/A) and recorded after 16-bit AD conversion at a sampling frequency of 100 kHz and a sampling number of 10^4^. The whole measurement process is controlled by a homemade software developed with LabVIEW (National Instruments, Austin, TX, USA).

## 3. Results and Discussion

### 3.1. Control of the Plug Motion and LAPS Measurement

**Figure 3:**
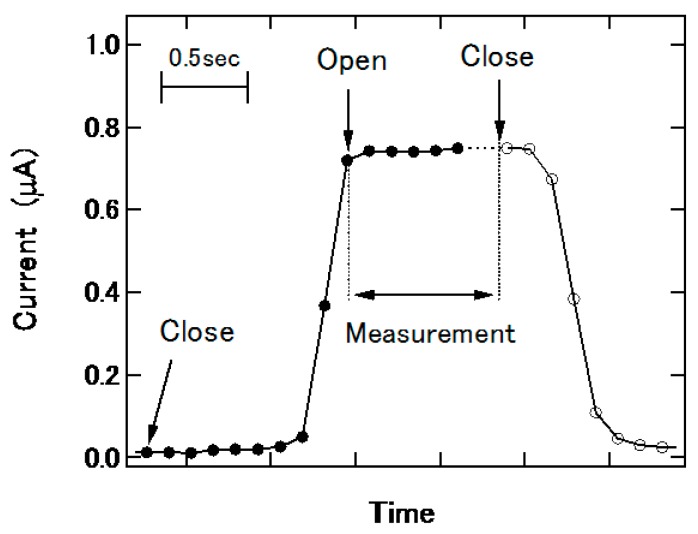
Detection of the plug by the photocurrent signal.

After applying a droplet at the inlet and closing the vent valve, the plug moves through the channel at a constant velocity. A bias voltage of −0.5 V is applied to the Ag/AgCl electrode with respect to the silicon substrate and the arrival of the plug at the electrode position is detected by monitoring the photocurrent signal as shown in Figure 3. The increase of the photocurrent was due to the arrival of the plug, which connected the Ag/AgCl electrode and the illuminated point of the sensor. When the photocurrent exceeds a predefined threshold, the vent valve is automatically opened so that the plug stays at the electrode position during the LAPS measurement, in which the photocurrent (I) is recorded as a function of the bias voltage (V). When the I-V curve is obtained, the vent valve is closed again and the plug is led to the outlet.

**Figure 4:**
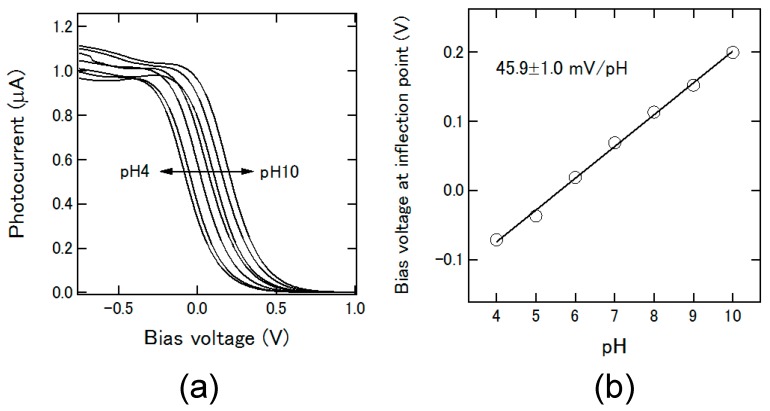
(**a**) I-V curves measured for plugs with different pH values; (**b**) Inflection points of I-V curves plotted as a function of pH.

Figure 4a shows an example of I-V curves obtained with the channel in Figure 1a for plugs of pH 4 to pH 10, each with a length of 2 mm and a volume of 1 μL. The bias voltage was swept in the range of −1.0 V to +1.0 V with a step width of 10 mV. In this series of experiments, different amounts of NaCl were added to each pH buffer (Titrisol, Merck, KGaA, Darmstadt, Germany), so that the total chloride concentration of the plug was always 0.05 M, in order to avoid the influence of the chloride sensitivity of the Ag/AgCl electrode. The inflection point of each I-V curve was calculated and plotted as a function of pH as shown in Figure 4b, in which a linear response with a pH sensitivity of 45.9 ± 1.0 mV/pH was observed.

In order to further reduce the volume of a plug, the height of the channel was reduced from 500 μm to 200 μm, while keeping the same width of 1 mm. A stable plug with a length of 2 mm and a volume of 400 nL could be reproducibly formed. The amplitude of the obtained photocurrent signal was almost the same as in the case of a 1 μL plug, as the contacting area of the plug with the sensing surface and the intensity of illumination were the same. The shift of the I-V curve (data not shown) was again linearly dependent on the pH value, with a pH sensitivity of 47.5 ± 1.9 mV/pH.

### 3.2. Merging Plugs and Differential Measurement

**Figure 5:**
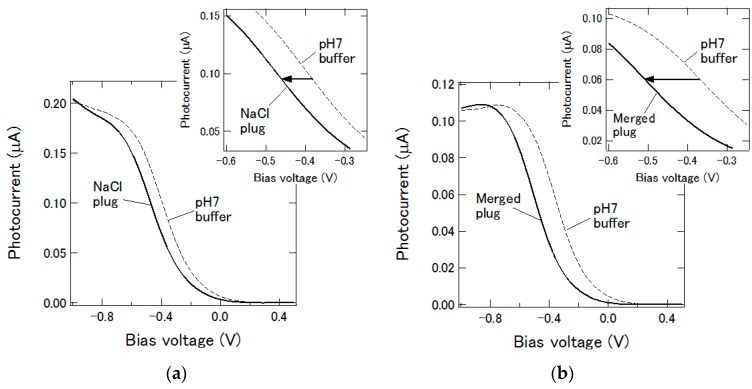
(**a**) I-V curves for the second plug measured in the upstream before merging; (**b**) I-V curves for the merged plug measured in the downstream.

In many assays, a test reagent is added to the sample to cause some reactions to be detected. The addition of enzyme to its substrate, the addition of antibody to antigen, or vice versa are commonly used in such tests. To mimic such a situation, two plugs were merged inside the microfluidic channel using the structure proposed in Reference [9] and the pH change was detected using the channel pattern in Figure 1b. The measurement areas in the upstream and the downstream of the chamber were simultaneously monitored by using two optical fibers illuminating these areas with two light beams modulated at different frequencies of 15 kHz and 14.9 kHz, respectively. By extracting each frequency component from the obtained photocurrent signal, the pH values at both positions could be independently determined [10]. Firstly, a solution with the total chloride concentration of 0.154 M was prepared by adding NaCl to 1 mM HCl solution. Then, 1 μL of this solution was introduced into the channel as the first plug, which stayed inside the merging chamber. The second plug, 1 μL of 0.154 M NaCl solution, was introduced into the channel and delivered to the first measurement area in the upstream, where the I-V curve in Figure 5a was obtained. The horizontal position of this curve was shifted by −76.8 mV with respect to that of a pH 7 buffer measured at the same position, and considering that the pH sensitivity at this position determined by a preliminary experiment was 51.5 ± 0.9 mV/pH, the pH value of the second plug was calculated to be 5.5. The second plug then entered the merging chamber and the merged plug of 2 μL was delivered to the measurement area in the downstream. The I-V curve for the merged plug is shown in Figure 5b, which is shifted by −145.9 mV with respect to that of a pH 7 buffer measured at the same position. Using the pH sensitivity value of 43.7 ± 1.1 mV/pH at this position, the pH value of the merged plug was calculated to be 3.7. This value was slightly higher than 3.3, calculated for 1 mM HCl after dilution to double. The result in this section confirms that the proposed device can be used for detecting a pH change induced by the addition of a plug of reagent to a plug of sample, which is expected to be applicable to various types of enzymatic and immuno-assays.

### 3.3. Plug Generation in the Channel

**Figure 6:**
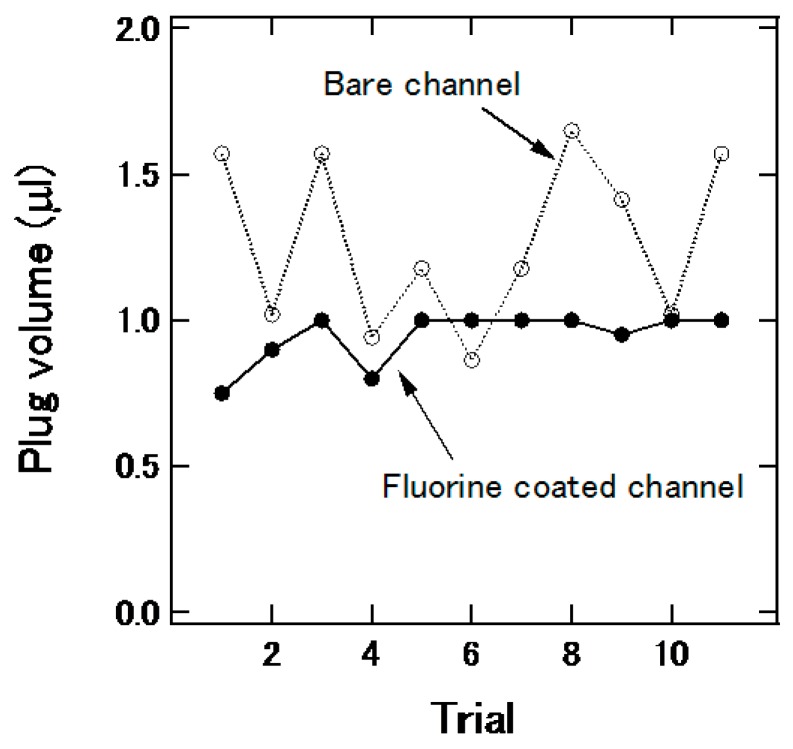
Effect of fluorine treatment of channel on the variation of plug volume.

In the channels shown in Figure 1a,b, the plug was manually prepared before measurement; however, for practical use, it is desired that the plug can be generated on a chip. Thus, plug generation using the channel structure in Figure 1c was examined. Typical air flow in the main channel and the injection speed of the sample were 1.67 μL/s and 3.0 μL/s, respectively. The channel was treated with a fluorine coating agent (FS-1060-2.0, Fluoro Technology Co., Ltd., Aichi, Japan) to make the surface hydrophobic. Figure 6 shows the effect of the treatment on the reproducibility of plug generation. The variation of the plug volume was suppressed by the fluorine coating.

### 3.4. Mixing of Two Plugs for Reaction

**Figure 7:**
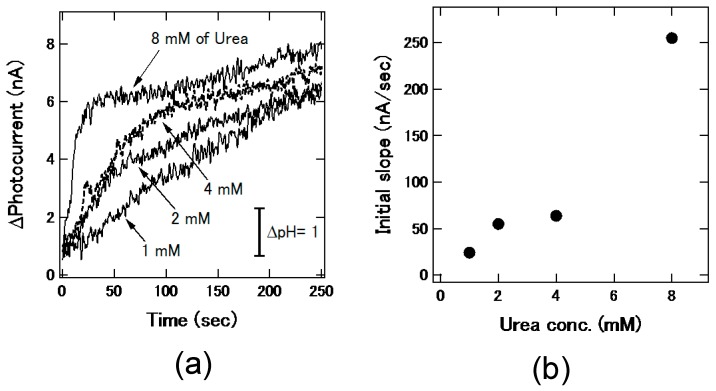
(**a**) Temporal change of photocurrent after merging a plug of urea solution and that of urease solution. The photocurrent response varied depending on concentrations of urea; (**b**) Initial slope of photocurrent change as a function of the urea concentration.

In the channel shown in Figure 1d, the sample chambers were filled with a solution of urea (U0631, Sigma-Aldrich, St. Louis, MO, USA) and that of urease from the Jack bean (U1500, Sigma-Aldrich, 15,000–50,000 units/g), the latter of which catalyzes the hydrolysis of the former. Both of urea and urease were dissolved in 5 mM phosphate buffered saline (PBS), and the concentration of urease was 2.8 g/L. The first plug from the chamber of the urea solution and the second plug from that of the urease solution were generated, and they were mixed in the merging chamber. After that, the merged plug moved to the sensing area at the downstream, and then the change of the pH value due to the enzymatic reaction in the mixed plug was observed as the change of the photocurrent. The photocurrent was recorded at a fixed bias voltage of −1.0 V. As demonstrated in past studies [6,7,8], the response of the sensor was fast enough to monitor the enzymatic reaction. Figure 7a shows the time-course of the photocurrent with different urea concentrations from 1 mM to 8 mM. Although the data contains noise due to the small change of the photocurrent, the photocurrent increased with the time after merging in all cases, which indicated the increase of the pH value due to the production of ammonium molecules [6]. In addition, the initial slopes of the photocurrent change in 0–25 s for 8 mM of urea and 0–50 s for other concentrations are shown in Figure 7b. The slope varied depending on the urea concentration. The plug generation, mixing, and sensing of the pH change of the plug on the chip were successfully demonstrated.

## 4. Conclusions

A plug-based microfluidic device was developed on the basis of a LAPS. The motion of the plug was pneumatically controlled so that the plug stayed at the electrode position during the LAPS measurement. A linear shift of the I-V curve depending on the pH value was observed for a plug with a volume down to 400 nL. Two successive plugs were merged inside the channel and the pH values before and after merging were successfully measured in the upstream and the downstream of the merging chamber. In addition, plug generation in the channel and detection of enzymatic reaction were also examined. A pH change caused by hydrolysis of urea catalyzed by urease was observed. Based on the light-addressability of LAPS, many detection points can be defined within the channel, and, therefore, the system is expected to be applicable to various kinds of assays where a small volume of samples are tested with reagents.
